# Structural Analysis of Retrovirus Assembly and Maturation

**DOI:** 10.3390/v14010054

**Published:** 2021-12-29

**Authors:** Anna-Sophia Krebs, Luiza M. Mendonça, Peijun Zhang

**Affiliations:** 1 Division of Structural Biology, Wellcome Trust Centre for Human Genetics, University of Oxford, Oxford OX3 7BN, UK; anna-sophia.krebs@lincoln.ox.ac.uk (A.-S.K.); luiza.montenegromendonca@strubi.ox.ac.uk (L.M.M.); 2 Electron Bio-Imaging Centre, Diamond Light Source, Harwell Science and Innovation Campus, Didcot OX11 0DE, UK; 3 Chinese Academy of Medical Sciences Oxford Institute, University of Oxford, Oxford OX3 7BN, UK

**Keywords:** HIV-1, maturation, capsid, cryoEM, cryoET, retroviruses, structure, assembly

## Abstract

Retroviruses have a very complex and tightly controlled life cycle which has been studied intensely for decades. After a virus enters the cell, it reverse-transcribes its genome, which is then integrated into the host genome, and subsequently all structural and regulatory proteins are transcribed and translated. The proteins, along with the viral genome, assemble into a new virion, which buds off the host cell and matures into a newly infectious virion. If any one of these steps are faulty, the virus cannot produce infectious viral progeny. Recent advances in structural and molecular techniques have made it possible to better understand this class of viruses, including details about how they regulate and coordinate the different steps of the virus life cycle. In this review we summarize the molecular analysis of the assembly and maturation steps of the life cycle by providing an overview on structural and biochemical studies to understand these processes. We also outline the differences between various retrovirus families with regards to these processes.

## 1. Introduction

Retroviruses are positive RNA viruses; many of those cause diseases of major importance to humans and domestic animals. Their name comes from their ability to reverse transcribe their genome and insert it into the host cell. The retrovirus family can be divided into several subfamilies based on their nucleic acid sequences and their life cycles. Alpha (e.g., Rous Sarcoma Virus—RSV), Beta (e.g., Human Endogenous Retrovirus K—HERV-K), Gamma (e.g., Murine Leukaemia Virus—MLV), Delta (e.g., Human T-cell Leukaemia Virus—HTLV), Epsilon-retroviruses (e.g., Walleye Dermal Sarcoma Virus—WDSV), Lentiviruses (e.g., Human Immunodeficiency Virus—HIV), and Spumaretroviruses (e.g., Human Foamy Virus—HFV). All subfamilies encode the open reading frames *gag*, *pol* and *env*, but differences in their genomic organization and additional regulatory proteins make each family unique.

## 2. Gag Is the Driver of Retroviral Assembly

**Figure 1:**
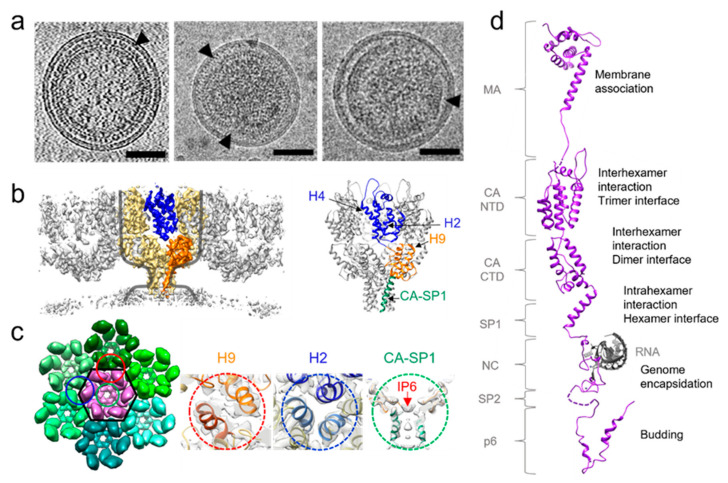
Retroviral immature lattice organization. (**a**) Tomographic slice of an HIV-1 (left), and cryoEM images of RSV (center) [15] and HTLV-1 (right) [15] virus-like particles, black arrowheads point to Gag lattice. Scale bars, 50 nm. (**b**) HIV-1 immature Gag structure, shown as cryoET subtomoram averaging map (left) and atomic model of the hexamer (right). One hexamer is highlighted in yellow in cryoET STA map within which one monomer is colored in blue (NTD) and orange (CTD and SP1). Grey outline shows the wine glass profile of the Gag hexamer [27]. (**c**) HIV-1 immature lattice organization in top view. Details of the dimeric, trimeric and hexameric interfaces are shown in gold, blue and green dashes, respectively. (**d**) HIV-1 Gag polyprotein domain organization. Original PDB accession codes: 1UPH (MA) [5], 7ASH (CA and SP1) [27], 1F6U (NC and SL2 RNA loop) [40], 2C55 (p6) [41].

The polyprotein Gag is the main driver of retrovirus assembly. Gag is synthetized as a long polyprotein upon the translation of the unspliced viral RNA (vRNA). Apart from Spumaviruses, Gag is cleaved by the viral protease during viral maturation to form the infectious and mature viral core. Gag consists of three conserved major protein domains: Matrix protein (MA), which targets Gag to the viral assembly sites, Capsid protein (CA), which is responsible for the multimerization necessary for assembly of both the immature and mature virus, and Nucleocapsid (NC), which binds the genomic RNA (gRNA) (Figure 1d).

Upon synthesis in the cytoplasm, Gag traffics to the viral assembly sites in an auto-inhibited conformation [1] to prevent early polymerization. These sites are the plasma membrane for alpha, delta, gamma, and lentiviruses. Betaretroviruses (with the exception of HERV-K) and spumaviruses have been described to assemble at internal cellular membranes [2,3]. The assembly site of epsilonretroviruses is still unknown. The myristoylation of Gag is semi-conserved between retroviruses, which has been observed in HIV, MLV, HTLV, and Bovine Leukaemia Virus (BLV). This post-translational modification is believed to be important for membrane targeting. Interestingly, betaretroviruses such as mouse mammary tumor virus (MMTV) and mason-pfizer monkey virus (MPMV) also have myristoylated Gag, but their assembly sites are at internal membranes. RSV, Equine infectious anemia virus (EIAV) and FV Gag are not myristoylated. For HIV-1, the binding of MA to PI(4,5)P2 (Phosphatidylinositol 4,5-bisphosphate) on the plasma membrane exposes the myristoyl moiety which is inserted into the inner leaflet of the plasma membrane [4,5]; however, this is not a conserved feature amongst retroviruses [6]. Fluorescence fluctuation spectroscopy findings suggest that different retroviruses have different requirements regarding Gag dimerization and oligomerization before being trafficked to the plasma membrane [7]. This discrepancy may also be explained by different affinities of Gag, particularly MA, to specific lipids in cellular membranes [6]. CryoEM data has shown large hexagonal assemblies consistent with Gag lattices in the cytoplasm of HIV infected cells [8].

Two copies of unspliced positive sense RNA genomes are incorporated into the virion, which act as a template for reverse transcription upon infection of a new cell. Due to limited space in the virion, this incorporation must be very specific. This high specificity is achieved by Gag recognizing the packaging signals in the 5′ UTR. The unspliced gRNA is first exported from the nucleus, recognized in the cytoplasm by Gag, and transported to the assembly sites [9]. The main recognition site in Gag is the CCHC-type zinc finger in NC. The initial interaction between the two genomes is guided by the stem loop 1 in the 5′UTR, with various other sites in the genome helping this interaction. The HIV-1 non-translating RNA dimerizes at the plasma membrane (PM), where it is stabilized by Gag and recruited into assembly complexes [10,11]. However, Gag must also bind to the RNA via other domains, as it has a higher affinity to RNA than the NC domain alone. The MA also has an RNA binding domain which overlaps with its PI(4,5)P2 binding site for membrane binding. This makes it possible to use cellular tRNAs to regulate membrane binding and to prevent early assembly inside the cell [12,13].

Gag is also responsible for recruiting the cellular machinery required to pinch off the nascent viral particles from the host cell: ESCRT (endosomal sorting complex required for transport). This recruitment is mediated by small peptide sequences in Gag called late domains. In HIV, this is achieved by the p6 domains, which binds the early ESCRT factors that recruits the rest of the complex needed for budding. Budding HIV-1 viruses have been studied by cellular cryoET, revealing abundant hemispherical budding profiles of Gag protein, with relatively few complete spherical buds linked to the plasma membrane via a membrane neck [8,14]. This indicates that the formation of the hemispherical bud is a kinetically slow intermediate, and that the completion of membrane fission and budding event is a fast process. The presence of filamentous actin was frequent at the budding sites; however, evidence of filamentous ESCRT complexes has so far not been observed by cellular cryoET [8,14]. No evidence of mature-like viruses bound to the plasma membrane has been found to date; at the same time, most viral particles found near cells are mature in morphology. This suggests that viral maturation is a remarkably fast process, and that viral protease activation happens concomitantly with the final stages of budding or shortly after. It is still unclear what triggers the activation of protease and subsequent cleavage of Gag. The newly budded immature virus is roughly spherical, with Gag strongly associated with the viral membrane through the MA domain, with a discontinuous spherical shell formed by CA-SP1 domain interactions. The RNA is also associated with the Gag shell, through interactions with the NC domain.

## 3. Immature Gag Lattice Structure

Apart from Spumaviruses, all retroviruses share a common morphology of the immature particle, with a dense, roughly spherical protein shell which stretches across approximately two-thirds of the internal surface of the virion (Figure 1a) [15]. The immature virus is formed exclusively by Gag hexamers that form a lattice through interactions of the CA domains as well as between SP1 or equivalent domains. An 8 nm spacing between hexamers is also a conserved feature of immature retroviral lattices [16,17,18,19]. However, a lattice consisting of only hexamers is mostly flat. To allow for curvature, it must incorporate holes or defects to fit onto a spherical surface [20]. The CA protein itself is divided into two subdomains: N-terminal (NTD) and C-terminal domains (CTD) separated by a flexible linker region. Despite low sequence conservation, the CA tertiary structure is highly conserved among retroviruses. The NTD generally consists of seven alpha helices, whereas the CTD is made up of four alpha-helices. These two domains are then joined by a flexible linker and oriented in such a way that they can form interactions to stabilize the hexamer [21]. The hexamer is the main building-block of the immature virus lattice. Intra-hexamer interactions are performed by a six-helix bundle (6HB) at the end of CTD and a spacer peptide downstream of capsid. Inter-hexamer interactions are performed by dimerization and trimerization of CA to form a lattice.

As the immature lattice is quite heterogenous in size and morphology, resolving its complete structure is difficult. Some domains of HIV-1 Gag were solved individually in earlier studies by X-ray crystallography and NMR [22,23,24]. Recently, the structure of HIV-1 CA CTD and the spacer peptide that recapitulates the immature lattice has been solved by X-ray crystallography [25]. For the structures of native Gag immature lattice assemblies, cryoET and subtomogram averaging (STA) provides a very powerful method [19,26,27,28,29], allowing for in situ structures to reach a resolution of ~3 Å [26]. These studies have shown that the hexameric assembly unit of immature CA lattice takes the shape of a wine glass, with the cup walls formed by the NTD, the cup bottom by the CTD, the stem by the 6HB formed by the last residues of CA and SP1 and the base by the amorphous NC/vRNA layer (Figure 1b) [30]. The neighbouring Gag hexamers are inter-connected to form a hexagonal lattice through trimeric interactions in the corners (blue circle). Three hexamers join, causing dimeric interactions (red circle) on the sides of the hexamer (cartoon overview Figure 1c). Helix 2 is responsible for the trimeric interactions between hexamers and the top of helix 4 in the NTD interacts with helices 5 and 6 in neighbouring hexamers (Figure 1c). In the CTD, the major homology region (MHR) is used for interactions within hexamers and the helix 9 contributes to the dimer interface between hexamers [18,31]. In contrast to the mature core, the Gag domains are organized in a linear fashion, meaning there are no contacts between NTDs and CTDs in immature lattices. Most viruses solved to date, such as HIV, RSV, EIAV and MLV, have the dimerization domain located in the CA CTD helix 9, while for HTLV-1 it seems to be in the NTD domain [17,18,19,32,33]. This unique feature may explain why HTLV-1 is the only retrovirus to display an immature lattice with straight facets [34].

A very important region within the immature lattice is the 6HB at the end of CA CTD and the beginning of SP1 (or a corresponding spacer peptide). This is the region where maturation inhibitors, such as Bevirimat (BVM) and PF46396 (PF96), bind [35,36]. In addition, a small molecule from the host cell, inositol hexaphosphate (IP6), was recently discovered to bind to the highly positive charged region at the top of 6HB and acts as a conserved assembly co-factor for different retroviruses, critical for the immature lattice assembly and viral infectivity [27,37]. The MHR, the loop between helix 9 and 10, and the beta-turn at the end of helix 11 further stabilize the 6HB. The 6HB could also be found in different retroviruses, such as MLV, EIAV and RSV [32,38]. NMR and molecular dynamic simulations have shown that this helix bundle is under a dynamic helix-coil equilibrium and that this is an important feature for optimal maturation and infectivity [39]. A second site compensatory mutation in the space peptide (T8I in HIV-1) in the BVM resistant mutant was shown to alter the dynamic property of 6HB and enable the formation of a fully extended stable 6HB connecting to the NC/vRNA layer [27]. Although all retroviral immature lattices solved to date share a hexameric immature lattice arrangement, the arrangement of CA NTD and its protein contacts differ between them, while the CA CTD interactions are highly conserved [16,17,18,19].

## 4. Retroviral Maturation Is a Finely Regulated Process

The budded immature particles are non-infectious. During, or shortly after budding, the viral protease is activated and starts cleaving the viral polyproteins into their individual constituents, which subsequently assemble into the infectious mature virion. The consequence of maturation is reflected in three aspects: at the sequence level, it is cleaved at all sites between structural proteins and spacer peptides by the protease; at the structure level, it undergoes structural rearrangements between CA subdomains and NC, vRNA and integrase (IN); at the architectural level, it converts from a spherical immature core only containing Gag hexamers to a mature conical shell containing CA hexamers and pentamers enclosing a condensed ribonucleoprotein complex (RNP).

The sequence and kinetics of cleavage is determined by the affinities between the cleavage sites and protease active site, as well as the accessibility of the cleavage sites. Any event that alters the sequence, rate or timing of maturation has drastic effects on viral morphology and infectivity [42]. In HIV-1, biochemical assays have shown that the first cleavage site within Gag to be processed is at the boundary of spacer peptide 1 (SP1) and NC. This cleavage releases NC and vRNA to condense into the RNP. The subsequent cleavages happen at the SP2-p6 and MA-CA boundaries, as well as at the NC-SP2 site. Finally, the last cleavage separates CA from SP1. This cleavage destabilizes the CA-SP1 6HB and was termed a maturation switch responsible for the large architectural rearrangement that converts the spherical immature assembly into a conical mature core [43].

The cleavage of viral polyproteins into individual domains is performed by the viral PR. The viral PR is itself part of the GagPol (or GagProPol, depending on the retroviral subfamily) polyprotein, present at a ratio of 1:20 to 1:10 in relation to the polyprotein Gag. The recruitment of GagPol to the viral assembly sites is thought to occur through the same Gag–Gag interactions that build the immature virus. Moreover, the viral PR is active only as a homodimer. It is not clear yet what is the trigger of viral PR activation, but it stands to reason that the first initial step in PR activation must be the dimerization of two PR domains within two GagPol polyproteins. PR dimerization may also be facilitated by the dimerization of RT and IN domains situated downstream of PR, as artificially enhancing or impairing RT and IN dimerization causes a corresponding effect on PR, with either premature or delayed activation [44,45,46]. In vitro cleavage studies have shown that a PR homodimer embedded in GagPol has much lower catalytic activity than ‘free’ PR homodimer. It is also suggested that the PR dimer in GagPol is constantly sampling different conformations and that the correct and active homodimer conformation is achieved only 5% of the time [47]. The initial cleavage events in maturation occur in an intramolecular fashion, where a GagPol-embedded PR dimer cleaves sites present in the same polyprotein that contains it. The initial sites to be cleaved in GagPol are on the SP1-NC boundary (also the initial cleavage in Gag) within an internal site in p6*, a domain of GagPol that sits upstream of PR and that is created by the frameshift event that leads to GagPol synthesis [48]. It is thought that p6* is an important spatiotemporal regulator of PR activation [49]. Accessory viral proteins such as Vif and Nef have also been described as regulators of PR activity [50,51]. The subsequent intramolecular cleavage occurs at the boundary of p6* and PR frees the PR N-terminal end. This is accompanied by an increase in PR catalytic activity, which is a consequence of better PR folding, particularly the formation of the four stranded beta-sheet that stabilizes the PR homodimer [52]. The cleavage of the GagPol domains downstream of PR, as well as the cleavage of Gag and other viral proteins, is thought to proceed in an intermolecular fashion [48]. Although it is not known what triggers PR activation, this is clearly a highly regulated (spatially and temporally) and sensitive event. Attempts to pause PR activation and synchronize maturation led to gross structural defects in the resulting viral particles, even though proteolytic cleavage of the polyproteins proceeded successfully once the PR pause was lifted [42]. This suggests that not only the cleavage events need to happen in the right sequence and rate, but also that maturation needs to be coordinated with other late viral replication events, probably assembly and budding.

The radical rearrangement of the virus architecture that most profoundly marks appropriate completion of HIV-1 maturation is the enclosure of the RNP inside a conical capsid. This affects the organization of multiple viral components: MA, CA, NC/vRNA, IN and Env. For HIV, recent cryoET findings have shown that MA trimers form a sparse and poorly ordered hexagonal lattice in the immature virus. Upon maturation, which curiously does not depend on proteolytic cleavage between MA-CA, this lattice becomes more ordered and tightly packed via MA highly basic region, with the inter-MA trimer interfaces completely different from the immature lattice [53]. The charge of the central pore in the hexamer of MA trimers also changes upon maturation, from basic-charged to neutral, which may have consequences on Env cytoplasmic tail interactions [53]. Binding of genomic RNA by the IN is critical for HIV-1 maturation [54]. Inhibition of IN-RNA interactions resulted in mislocalization of the RNP to the exterior of the mature capsid, as shown by the effects of HIV-1 class II IN mutations, as well as of Allosteric IN inhibitors (ALLINIs) on HIV-1 maturation [55]. It has been reported that Env arrangement is also affected during maturation, with Env trimers changing from a scattered and low-mobile state in the immature particle to a highly mobile and clustered state in the mature virus [56].

There have been three hypotheses for how the architectural maturation (the switch from spherical immature lattice to a conical mature lattice) takes place: displacive transition, de novo assembly, and sequential combination of displacive nucleation followed by de novo assembly. The displacive model considers that the CA rearrangement occurs concomitantly with maturation, and that a portion of the cleaved lattice rolls away from the membrane while associated with the condensing RNP to form a mature conical core [57]. The displacive model is inspired by in vitro studies on non-diffusional transitions of tubular CA-NC assemblies when PR is added to the system [58]. The de novo assembly model postulates that, upon cleavage, the immature lattice is completely disassembled and a subset of the now soluble CA proteins re-oligomerize to form the mature core. This model is supported by the observation that the CA subdomain re-orientation and novel protein-protein contacts necessary to transition from the immature lattice to the mature lattice cannot be accommodated in the whole virus lattice due to spatial constraints. The third model is a combination of the previous two and suggests that the initial mature lattice nucleation step happens by a displacive mechanism that has a large contribution from the RNP condensation. The expansion of the mature lattice into an enclosed core happens by a de novo mechanism starting from the previously displacive-originated mature lattice. This has been supported by in vitro maturation studies combining parallel biochemical assays, cryoEM and computation modelling [59].

There are also competing hypotheses on the directionality of the HIV-1 mature core assembly. It was initially proposed that the core assembly proceeded from the base to the tip of the cone. This was supported by some cryoET observations: the HIV cone has a consistent cone angle between 18–24°; the cone wide base has a consistent 11 nm distance to the viral membrane; HIV RNP is located at the bottom of the conical core. The authors postulated that the RNP had a big role in nucleating the CA mature lattice and that the cone growth started there until it reached the other end of the viral membrane. It was also observed that the tip end of the cone frequently had a hole, postulated as a cone-closing defect [60]. A competing hypothesis proposes the opposite, that the core assembles from the tip to the base of the cone. This is also supported by cryoET observations, namely that the HIV cone spanned the whole diameter of the viral particle, even when viral particles varied in diameter. As such, a cone that grew from the tip towards the wide end would grow until it reached the opposing side of the viral membrane, at which point the membrane resistance would force the growing facet to bend (by the insertion of CA pentamers) until it closed at the base [61]. The third hypothesis for HIV-1 core assembly stems from the combination of cryoET and computer simulations of the nonequilibrium growth of elastic sheets. This third model proposed that core formation proceeds from a mature CA lattice sheet with the tendency to curve. This intrinsic curving propensity is given by the nature of the CA unit, which can be approximated to a tapered prism-shaped 3D subunit. The growth of the core started with the polymerization of a curved lattice sheet, in which the insertion of pentamers was necessary in order to resolve high-curvature regions. Eventually, the growing CA sheet would curl and meet itself on the other end, closing the core. By modulating the simulation parameters, this model could recapitulate all shapes found in retroviral capsids, from polyhedral, to cones and tubes. It also recapitulated many core defects found in conical cores, such as jelly-rolls and closing gaps, both at the side of the cone and at the narrow tip of the core [62,63].

## 5. Mature Core Lattice Structure

**Figure 2:**
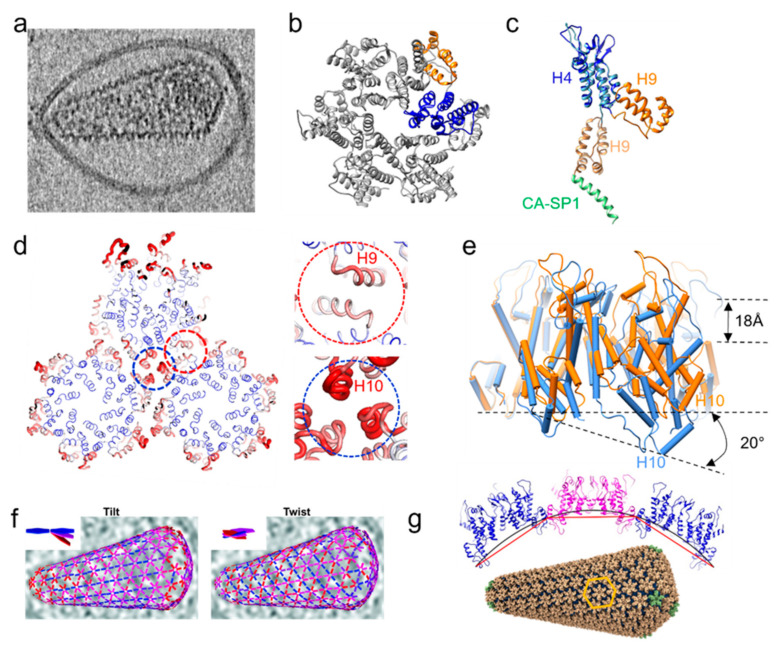
HIV-1 mature capsid organization. (**a**) Tomographic slice of a mature HIV-1 particle with a capsid cone [76]. (**b**) Atomic model of an HIV-1 mature CA hexamer derived from tubular assemblies (PDB 6SKK [77]). One CA monomer is coloured in blue (NTD) and orange (CTD). (**c**) Comparison of immature CA-SP1 (lighter shade) and mature CA structure, aligned at the NTD (blue). (**d**) HIV-1 mature lattice organization in top view. The width and color of the sausage are directly proportional to the B-factor, from blue (−30) to red (−100). Details of the dimeric and trimeric interfaces are shown in red and blue dashes, respectively. (**e**) Intrinsic curvature of the mature CA hexamer by superposition of a planar CA hexamer (orange, PDB 4XFX [78]) and a highly curved hexamer (blue, PDB 6SKK [77]). (**f**) Tilt (left) and twist (right) angles between hexamers within a single core. Insets show a schematic illustration of tilt and twist angles [76]. The tilt/twist angle is indicated by the colour of the connecting lines between hexamer positions, from blue (less tilt along the long axis of the core) to red (more tilt along the circumference). (**g**) All-atom atomic model of theHIV-1 conical core (bottom) and a cross-section of three hexamers along the curved direction (top). The black line illustrates continuous curvature of the lattice given by both intra and inter-hexamer curvature, while the red line illustrates discrete curvature given by inter-hexamer curvature alone [77]. The lattice unit is marked with orange hexagon; pentamers shown in green [72].

Upon maturation, a subset of the CA protein assembles to form the mature core (Figure 2a). The mature core is a metastable structure responsible for protecting the viral genome from detection by cytoplasmic host innate immunity sensors, as well as to provide a compartment for reverse transcription initiation. The capsid core must also be able to uncoat to release the reverse transcribed genome for integration in the host genome. The capsid further acts as a docking platform for host proteins to facilitate the transport of virus core through the cytoplasm into the nucleus [64,65,66]. Many host restriction factors also recognize the mature capsid surface lattice and evoke inhibitory actions [67,68,69,70]. 

The mature retrovirus capsid core follows the fullerene geometry model. It is predominantly made up of hexamers (Figure 2b) and incorporates a few pentamers. These pentamers are necessary, as they allow for high curvature in the lattice. For lentiviruses, this core has a conical shape. For alpha, beta, gamma, and deltaretroviruses, this core is polyhedral or cylindrical in shape. The overall core shape is determined by the placement of the pentamers in the core. In a conical core, this is done by a partition of seven pentamers in the broad cone end, and five at the narrow end. A 6-6 partition leads to a cylindrical core, while a randomly distributed partition creates a polyhedron. There are also viruses which incorporate more than 12 pentamers, such as MLV. MLV has multi-layered or multiple cores as it incorporates almost all cleaved CA molecules, and the core is less tightly packed. The various MLV core morphologies require up to 24 pentamers but they are remarkably similar in structure to their neighbouring hexamers [17]. HTLV-1 mature cores are very often incomplete, which may explain the remarkably low infectivity of this retrovirus [71].

In HIV-1, the mature lattice has a thinner appearance (4 nm thick) in the cross section in comparison with the immature one (14 nm thick from CA NTD to NC/vRNA). The hexamer–hexamer spacing is larger than in the immature lattice, 10 nm instead of 8 nm. The key differences between immature and mature hexamers are the orientations of NTD and CTD and the intermolecular NTD–CTD contacts that stabilize the mature hexamer (Figure 2b,c). For the mature lattice assembly, the CTD is the main stabilizing domain between hexamers, specifically the 2-fold interface formed by helix 9 and the 3-fold interface formed by helices 10 and 11 (Figure 2d) [72,73]. The dimer interface appears stable and less flexible than the trimer interface, as suggested by the lower B-factor (Figure 2d). An important interface is formed by conserved positively charged residues at the center of the NTD hexamer. This position is reported to bind IP6 in an analogous manner as the positively charged ring found in the CTD at the top of 6HB in the immature lattice [37,74]. In HIV-1, above the mature hexamer center lies a beta-hairpin described to adopt different conformations depending on pH [75]. These conformations translate into different accessibilities to the central CA hexamer channel and have been hypothesized to be an important regulator of capsid core permeability to nucleotides necessary for reverse transcription of the viral genome [75].

The pentameric structure recently determined from native capsid by cryoET and subtomogram averaging is different from the previous crystal structure of the cross-linked pentamer [72,76]. Its NTDs are rotated by approximately 19 degrees compared to its hexameric counterpart. In doing so, it excludes helix 3 from the interface and forms a 10-helix bundle with its neighbouring hexamer instead. Moreover, the binding site for host factors and small molecules such as PF74 is more open at the pentamer NTD-CTD interface in comparison to the hexameric one [76]. The five arginine residues (R18) at the center of the pentamer in HIV CA (or the corresponding residue K17 in RSV CA) have been proposed to regulate the transition between hexamer and pentamer by balancing its electrostatic destabilization with stabilizing the lattice [38,79].

The CA domain of different viruses has very low sequence conservation; nonetheless, there is strong structural conservation between the CA proteins of different retroviruses [21]. This structurally conserved protein has a remarkable ability to accommodate different lattice curvatures. Recent cryoEM studies have shown that this ability is enabled by two features: the different tilt and twist orientations between CA hexamers [76], and more prominently by intrinsic curvatures of the hexamer itself (Figure 2e), enabled by the flexible linker between CA NTD and CTD [77] (Figure 2f,g). This curvature was also observed in the mature RSV capsid-like particles, as RSV has a highly variable core. This flexibility can adapt to the different requirements of the hexamers at various curvatures and a more random distribution of pentamers [38,80]. The curvature of the RSV capsid was thought to be derived from variable inter-capsomer interfaces; however, more recent structure data suggest the capsomers are intrinsically curved [38,77].

## 6. Future Perspective

Even with all the recent structural findings on the replication cycle of retroviruses, there are still many poorly understood processes. It is still unclear where Gag initially dimerizes, how it is transported to the assembly sites and how exactly maturation is triggered. The pathway of how the virus transitions from the immature to the mature capsid is also not completely understood. In addition, HIV-1 has served as the model organism to understand these concepts. Our understanding of other types of retroviruses has been catching up in recent years in terms of their mature and immature capsid architecture. Nonetheless, there is still much to be done to understand exactly how each virus regulates its assembly and maturation. Novel technical advances, such as correlative light and electron microscopy (CLEM) [81,82,83,84,85], cryoFIB lamella [86,87] cellular tomography [83,86], integrative imaging [83,88,89], and computational advances on subtomogram averaging [26,90] will play an important role filling these knowledge gaps in the near future.

## References

[1] Sundquist W.I., Kräusslich H.-G. (2012). HIV-1 Assembly, Budding, and Maturation. Cold Spring Harb. Perspect. Med..

[2] Flügel R.M., Pfrepper K.I. (2003). Proteolytic processing of foamy virus Gag and Pol proteins. Curr. Top. Microbiol. Immunol..

[3] Garcia-Montojo M., Doucet-O’Hare T., Henderson L., Nath A. (2018). Human endogenous retrovirus-K (HML-2): A comprehensive review. Crit. Rev. Microbiol..

[4] Bussienne C., Marquet R., Paillart J.C., Bernacchi S. (2021). Post-Translational Modifications of Retroviral HIV-1 Gag Precursors: An Overview of Their Biological Role. Int. J. Mol. Sci..

[5] Tang C., Loeliger E., Luncsford P., Kinde I., Beckett D., Summers M.F. (2004). Entropic switch regulates myristate exposure in the HIV-1 matrix protein. Proc. Natl. Acad. Sci. USA.

[6] Inlora J., Collins D.R., Trubin M.E., Chung J.Y.J., Ono A. (2014). Membrane Binding and Subcellular Localization of Retroviral Gag Proteins Are Differentially Regulated by MA Interactions with Phosphatidylinositol-(4,5)-Bisphosphate and RNA. mBio.

[7] Martin J.L., Mendonça L.M., Angert I., Mueller J.D., Zhang W., Mansky L.M. (2017). Disparate Contributions of Human Retrovirus Capsid Subdomains to Gag-Gag Oligomerization, Virus Morphology, and Particle Biogenesis. J. Virol..

[8] Woodward C.L., Cheng S.N., Jensen G.J. (2015). Electron Cryotomography Studies of Maturing HIV-1 Particles Reveal the Assembly Pathway of the Viral Core. J. Virol..

[9] Mailler E., Bernacchi S., Marquet R., Paillart J.C., Vivet-Boudou V., Smyth R.P. (2016). The life-cycle of the HIV-1 gag–RNA complex. Viruses.

[10] Chen J., Liu Y., Wu B., Nikolaitchik O.A., Mohan P.R., Chen J., Pathak V.K., Hu W.-S. (2020). Visualizing the translation and packaging of HIV-1 full-length RNA. Proc. Natl. Acad. Sci. USA.

[11] Chen J., Rahman S.A., Nikolaitchik O.A., Grunwald D., Sardo L., Burdick R.C., Plisov S., Liang E., Tai S., Pathak V.K. (2016). HIV-1 RNA genome dimerizes on the plasma membrane in the presence of Gag protein. Proc. Natl. Acad. Sci. USA.

[12] Kutluay S.B., Zang T., Blanco-Melo D., Powell C., Jannain D., Errando M., Bieniasz P.D. (2014). Global changes in the RNA binding specificity of HIV-1 gag regulate virion genesis. Cell.

[13] Todd G.C., Duchon A., Inlora J., Olson E.D., Musier-Forsyth K., Ono A. (2017). Inhibition of HIV-1 Gag-membrane interactions by specific RNAs. RNA.

[14] Carlson L.A., de Marco A., Oberwinkler H., Habermann A., Briggs J.A.G., Kräusslich H.G., Grünewald K. (2010). Cryo electron tomography of native HIV-1 budding sites. PLoS Pathog..

[15] Martin J.L., Cao S., Maldonado J.O., Zhang W., Mansky L.M. (2016). Distinct Particle Morphologies Revealed through Comparative Parallel Analyses of Retrovirus-Like Particles. J. Virol..

[16] Bharat T.A.M., Castillo Menendez L.R., Hagen W.J.H., Lux V., Igonet S., Schorb M., Schur F.K.M., Kräusslich H.-G., Briggs J.A.G. (2014). Cryo-electron microscopy of tubular arrays of HIV-1 Gag resolves structures essential for immature virus assembly. Proc. Natl. Acad. Sci. USA.

[17] Qu K., Glass B., Dolezal M., Schur F.K.M., Murciano B., Rein A., Rumlová M., Ruml T., Kräusslich H.G., Briggs J.A.G. (2018). Structure and architecture of immature and mature murine leukemia virus capsids. Proc. Natl. Acad. Sci. USA.

[18] Schur F.K.M., Hagen W.J.H., Rumlová M., Ruml T., Müller B., Kraüsslich H.G., Briggs J.A.G. (2015). Structure of the immature HIV-1 capsid in intact virus particles at 8.8 Å resolution. Nature.

[19] Schur F.K.M., Obr M., Hagen W.J.H., Wan W., Jakobi A.J., Kirkpatrick J.M., Sachse C., Kräusslich H.-G., Briggs J.A.G. (2016). An atomic model of HIV-1 capsid-SP1 reveals structures regulating assembly and maturation. Science.

[20] Briggs J.A.G., Kräusslich H.G. (2011). The molecular architecture of HIV. J. Mol. Biol..

[21] Bharat T.A.M., Davey N.E., Ulbrich P., Riches J.D., De Marco A., Rumlova M., Sachse C., Ruml T., Briggs J.A.G. (2012). Structure of the immature retroviral capsid at 8Å resolution by cryo-electron microscopy. Nature.

[22] Kelly B.N., Howard B.R., Wang H., Robinson H., Sundquist W.I., Hill C.P. (2006). Implications for viral capsid assembly from crystal structures of HIV-1 Gag(1-278) and CA(N)(133-278). Biochemistry.

[23] Morellet N., Druillennec S., Lenoir C., Bouaziz S., Roques B.P. (2005). Helical structure determined by NMR of the HIV-1 (345-392)Gag sequence, surrounding p2: Implications for particle assembly and RNA packaging. Protein Sci..

[24] Tang C., Ndassa Y., Summers M.F. (2002). Structure of the N-terminal 283-residue fragment of the immature HIV-1 Gag polyprotein. Nat. Struct. Biol..

[25] Wagner J.M., Zadrozny K.K., Chrustowicz J., Purdy M.D., Yeager M., Ganser-Pornillos B.K., Pornillos O. (2016). Crystal structure of an HIV assembly and maturation switch. Elife.

[26] Himes B.A., Zhang P. (2018). emClarity: Software for high-resolution cryo-electron tomography and subtomogram averaging. Nat. Methods.

[27] Mendonça L., Sun D., Ning J., Liu J., Kotecha A., Olek M., Frosio T., Fu X., Himes B.A., Kleinpeter A.B. (2021). CryoET structures of immature HIV Gag reveal six-helix bundle. Commun. Biol..

[28] Turoňová B., Schur F.K.M., Wan W., Briggs J.A.G. (2017). Efficient 3D-CTF correction for cryo-electron tomography using NovaCTF improves subtomogram averaging resolution to 3.4Å. J. Struct. Biol..

[29] Wright E.R., Schooler J.B., Ding H.J., Kieffer C., Fillmore C., Sundquist W.I., Jensen G.J. (2007). Electron cryotomography of immature HIV-1 virions reveals the structure of the CA and SP1 Gag shells. EMBO J..

[30] Campbell S., Fisher R.J., Towler E.M., Fox S., Issaq H.J., Wolfe T., Phillips L.R., Rein A. (2001). Modulation of HIV-like particle assembly in vitro by inositol phosphates. Proc. Natl. Acad. Sci. USA.

[31] Novikova M., Zhang Y., Freed E.O., Peng K. (2019). Multiple Roles of HIV-1 Capsid during the Virus Replication Cycle. Virol. Sin..

[32] Dick R.A., Xu C., Morado D.R., Kravchuk V., Ricana C.L., Lyddon T.D., Broad A.M., Feathers J.R., Johnson M.C., Vogt V.M. (2020). Structures of immature EIAV Gag lattices reveal a conserved role for IP6 in lentivirus assembly. PLoS Pathog..

[33] Martin J.L., Mendonça L.M., Marusinec R., Zuczek J., Angert I., Blower R.J., Mueller J.D., Perilla J.R., Zhang W., Mansky L.M. (2018). Critical Role of the Human T-Cell Leukemia Virus Type 1 Capsid N-Terminal Domain for Gag-Gag Interactions and Virus Particle Assembly. J. Virol..

[34] Maldonado J.O., Cao S., Zhang W., Mansky L.M. (2016). Distinct Morphology of Human T-Cell Leukemia Virus Type 1-Like Particles. Viruses.

[35] Keller P.W., Adamson C.S., Heymann J.B., Freed E.O., Steven A.C. (2011). HIV-1 maturation inhibitor bevirimat stabilizes the immature Gag lattice. J. Virol..

[36] Spearman P. (2016). HIV-1 Gag as an Antiviral Target: Development of Assembly and Maturation Inhibitors. Curr. Top. Med. Chem..

[37] Dick R.A., Zadrozny K.K., Xu C., Schur F.K.M., Lyddon T.D., Ricana C.L., Wagner J.M., Perilla J.R., Ganser-Pornillos B.K., Johnson M.C. (2018). Inositol phosphates are assembly co-factors for HIV-1. Nature.

[38] Obr M., Ricana C.L., Nikulin N., Feathers J.-P.R., Klanschnig M., Thader A., Johnson M.C., Vogt V.M., Schur F.K.M., Dick R.A. (2021). Structure of the mature Rous sarcoma virus lattice reveals a role for IP6 in the formation of the capsid hexamer. Nat. Commun..

[39] Wang M., Quinn C.M., Perilla J.R., Zhang H., Shirra R., Hou G., Byeon I.-J., Suiter C.L., Ablan S., Urano E. (2017). Quenching protein dynamics interferes with HIV capsid maturation. Nat. Commun..

[40] Amarasinghe G.K., De Guzman R.N., Turner R.B., Chancellor K.J., Wu Z.R., Summers M.F. (2000). NMR structure of the HIV-1 nucleocapsid protein bound to stem-loop SL2 of the psi-RNA packaging signal. Implications for genome recognition. J. Mol. Biol..

[41] Fossen T., Wray V., Bruns K., Rachmat J., Henklein P., Tessmer U., Maczurek A., Klinger P., Schubert U. (2005). Solution structure of the human immunodeficiency virus type 1 p6 protein. J. Biol. Chem..

[42] Mattei S., Anders M., Konvalinka J., Kräusslich H.-G., Briggs J.A.G., Müller B., Krausslich H.-G., Briggs J.A.G., Muller B. (2014). Induced Maturation of Human Immunodeficiency Virus. J. Virol..

[43] Mattei S., Tan A., Glass B., Müller B., Kräusslich H.-G., Briggs J.A.G. (2018). High-resolution structures of HIV-1 Gag cleavage mutants determine structural switch for virus maturation. Proc. Natl. Acad. Sci. USA.

[44] Hoyte A.C., Jamin A.V., Koneru P.C., Kobe M.J., Larue R.C., Fuchs J.R., Engelman A.N., Kvaratskhelia M. (2017). Resistance to pyridine-based inhibitor KF116 reveals an unexpected role of integrase in HIV-1 Gag-Pol polyprotein proteolytic processing. J. Biol. Chem..

[45] Sadiq S.K., Mirambeau G., Meyerhans A. (2018). Equilibrium Model of Drug-Modulated GagPol-Embedded HIV-1 Reverse Transcriptase Dimerization to Enhance Premature Protease Activation. AIDS Res. Hum. Retrovir..

[46] Sudo S., Haraguchi H., Hirai Y., Gatanaga H., Sakuragi J.-i., Momose F., Morikawa Y. (2013). Efavirenz enhances HIV-1 gag processing at the plasma membrane through Gag-Pol dimerization. J. Virol..

[47] Tang C., Louis J.M., Aniana A., Suh J.Y., Clore G.M. (2008). Visualizing transient events in amino-terminal autoprocessing of HIV-1 protease. Nature.

[48] Pettit S.C., Everitt L.E., Choudhury S., Dunn B.M., Kaplan A.H. (2004). Initial cleavage of the human immunodeficiency virus type 1 GagPol precursor by its activated protease occurs by an intramolecular mechanism. J. Virol..

[49] Ludwig C., Leiherer A., Wagner R. (2008). Importance of Protease Cleavage Sites within and Flanking Human Immunodeficiency Virus Type 1 Transframe Protein p6* for Spatiotemporal Regulation of Protease Activation. J. Virol..

[50] Bardy M., Gay B., Pébernard S., Chazal N., Courcoul M., Vigne R., Decroly E., Boulanger P. (2001). Interaction of human immunodeficiency virus type 1 Vif with Gag and Gag-Pol precursors: Co-encapsidation and interference with viral protease-mediated Gag processing. J. Gen. Virol..

[51] Mendonça L.M., Poeys S.C., Abreu C.M., Tanuri A., Costa L.J. (2014). HIV-1 Nef inhibits Protease activity and its absence alters protein content of mature viral particles. PLoS ONE.

[52] Louis J.M., Clore G.M., Gronenborn A.M. (1999). Autoprocessing of HIV-1 protease is tightly coupled to protein folding. Nat. Struct. Biol..

[53] Qu K., Ke Z., Zila V., Anders-Össwein M., Glass B., Mücksch F., Müller R., Schultz C., Müller B., Kräusslich H.G. (2021). Maturation of the matrix and viral membrane of HIV-1. Science.

[54] Kessl J., Kutluay S., Townsend D., Rebensburg S., Slaughter A., Larue R., Shkriabai N., Bakouche N., Fuchs J., Bieniasz P. (2016). HIV-1 Integrase Binds the Viral RNA Genome and Is Essential during Virion Morphogenesis. Cell.

[55] Jurado K., Wang H., Slaughter A., Feng L., Kessl J., Koh Y., Wang W., Ballandras-Colas A., Patel P., Fuchs J. (2013). Allosteric integrase inhibitor potency is determined through the inhibition of HIV-1 particle maturation. Proc. Natl. Acad. Sci. USA.

[56] Chojnacki J., Staudt T., Glass B., Bingen P., Engelhardt J., Anders M., Schneider J., Müller B., Hell S.W., Kräusslich H.G. (2012). Maturation-dependent HIV-1 surface protein redistribution revealed by fluorescence nanoscopy. Science.

[57] Frank G.A., Narayan K., Bess J.W., Del Prete G.Q., Wu X., Moran A., Hartnell L.M., Earl L.A., Lifson J.D., Subramaniam S. (2015). Maturation of the HIV-1 core by a non-diffusional phase transition. Nat. Commun..

[58] Meng X., Zhao G., Yufenyuy E., Ke D., Ning J., DeLucia M., Ahn J., Gronenborn A.M., Aiken C., Zhang P. (2012). Protease Cleavage Leads to Formation of Mature Trimer Interface in HIV-1 Capsid. PLoS Pathog..

[59] Ning J., Erdemci-Tandogan G., Yufenyuy E.L., Wagner J., Himes B.A., Zhao G., Aiken C., Zandi R., Zhang P. (2016). In vitro protease cleavage and computer simulations reveal the HIV-1 capsid maturation pathway. Nat. Commun..

[60] Benjamin J., Ganser-Pornillos B.K., Tivol W.F., Sundquist W.I., Jensen G.J. (2005). Three-dimensional Structure of HIV-1 Virus-like Particles by Electron Cryotomography. J. Mol. Biol..

[61] Briggs J.A.G., Grünewald K., Glass B., Förster F., Kräusslich H.G., Fuller S.D. (2006). The Mechanism of HIV-1 Core Assembly: Insights from Three-Dimensional Reconstructions of Authentic Virions. Structure.

[62] Levandovsky A., Zandi R. (2009). Nonequilibirum assembly, retroviruses, and conical structures. Phys. Rev. Lett..

[63] Yu Z., Dobro M.J., Woodward C.L., Levandovsky A., Danielson C.M., Sandrin V., Shi J., Aiken C., Zandi R., Hope T.J. (2013). Unclosed HIV-1 Capsids Suggest a Curled Sheet Model of Assembly. J. Mol. Biol..

[64] Liu C., Perilla J.R., Ning J., Lu M., Hou G., Ramalho R., Himes B.A., Zhao G., Bedwell G.J., Byeon I.-J. (2016). Cyclophilin A stabilizes the HIV-1 capsid through a novel non-canonical binding site. Nat. Commun..

[65] Ning J., Zhong Z., Fischer D.K., Harris G., Watkins S.C., Ambrose Z., Zhang P. (2018). Truncated CPSF6 Forms Higher-Order Complexes That Bind and Disrupt HIV-1 Capsid. J. Virol..

[66] Zhong Z., Ning J., Boggs E.A., Jang S., Wallace C., Telmer C., Bruchez M.P., Ahn J., Engelman A.N., Zhang P. (2021). Cytoplasmic CPSF6 Regulates HIV-1 Capsid Trafficking and Infection in a Cyclophilin A-Dependent Manner. mBio.

[67] Alvarez F.J.D., He S., Perilla J.R., Jang S., Schulten K., Engelman A.N., Scheres S.H.W., Zhang P. (2017). CryoEM structure of MxB reveals a novel oligomerization interface critical for HIV restriction. Sci. Adv..

[68] Wilbourne M., Zhang P. (2021). Visualizing HIV-1 Capsid and Its Interactions with Antivirals and Host Factors. Viruses.

[69] Zhang W., Mendonça L.M., Mansky L.M. (2018). The Retrovirus Capsid Core. Subcell. Biochem..

[70] Zhao G., Ke D., Vu T., Ahn J., Shah V.B., Yang R., Aiken C., Charlton L.M., Gronenborn A.M., Zhang P. (2011). Rhesus TRIM5α Disrupts the HIV-1 Capsid at the Inter-Hexamer Interfaces. PLoS Pathog..

[71] Meissner M.E., Mendonça L.M., Zhang W., Mansky L.M. (2017). Polymorphic Nature of Human T-Cell Leukemia Virus Type 1 Particle Cores as Revealed through Characterization of a Chronically Infected Cell Line. J. Virol..

[72] Ni T., Zhu Y., Yang Z., Xu C., Chaban Y., Nesterova T., Ning J., Böcking T., Parker M., Monnie C. (2021). Structure of Native HIV-1 Cores and Their Interactions with IP6 and CypA. Sci. Adv..

[73] Zhao G., Perilla J.R., Yufenyuy E.L., Meng X., Chen B., Ning J., Ahn J., Gronenborn A.M., Schulten K., Aiken C. (2013). Mature HIV-1 capsid structure by cryo-electron microscopy and all-atom molecular dynamics. Nature.

[74] Mallery D.L., Marquez C.L., McEwan W.A., Dickson C.F., Jacques D.A., Anandapadamanaban M., Bichel K., Towers G.J., Saiardi A., Bocking T. (2018). IP6 is an HIV pocket factor that prevents capsid collapse and promotes DNA synthesis. eLife.

[75] Jacques D.A., McEwan W.A., Hilditch L., Price A.J., Towers G.J., James L.C. (2016). HIV-1 uses dynamic capsid pores to import nucleotides and fuel encapsidated DNA synthesis. Nature.

[76] Mattei S., Glass B., Hagen W.J.H., Kräusslich H.-G., Briggs J.A.G. (2016). The structure and flexibility of conical HIV-1 capsids determined within intact virions. Science.

[77] Ni T., Gerard S., Zhao G., Dent K., Ning J., Zhou J., Shi J., Anderson-Daniels J., Li W., Jang S. (2020). Intrinsic curvature of the HIV-1 CA hexamer underlies capsid topology and interaction with cyclophilin A. Nat. Struct. Mol. Biol..

[78] Gres A.T., Kirby K.A., Kewalramani V.N., Tanner J.J., Pornillos O., Sarafianos S.G. (2015). X-ray crystal structures of native HIV-1 capsid protein reveal conformational variability. Science.

[79] Pornillos O., Ganser-Pornillos B.K., Yeager M. (2011). Atomic-level modelling of the HIV capsid. Nature.

[80] Acton O., Grant T., Nicastro G., Ball N.J., Goldstone D.C., Robertson L.E., Sader K., Nans A., Ramos A., Stoye J.P. (2019). Structural basis for Fullerene geometry in a human endogenous retrovirus capsid. Nat. Commun..

[81] Fu X., Ning J., Zhong Z., Ambrose Z., Watkins S.C., Zhang P. (2019). AutoCLEM: An Automated Workflow for Correlative Live-Cell Fluorescence Microscopy and Cryo-Electron Tomography. Sci. Rep..

[82] Jun S., Ke D., Debiec K., Zhao G., Meng X., Ambrose Z., Gibson G.A., Watkins S.C., Zhang P. (2011). Direct visualization of HIV-1 with correlative live-cell microscopy and cryo-electron tomography. Structure.

[83] Mendonça L., Howe A., Gilchrist J.B., Sheng Y., Sun D., Knight M.L., Zanetti-Domingues L.C., Bateman B., Krebs A.-S., Chen L. (2021). Correlative multi-scale cryo-imaging unveils SARS-CoV-2 assembly and egress. Nat. Commun..

[84] Tao C.-L., Liu Y.-T., Sun R., Zhang B., Qi L., Shivakoti S., Tian C.-L., Zhang P., Lau P.-M., Zhou Z.H. (2018). Differentiation and Characterization of Excitatory and Inhibitory Synapses by Cryo-electron Tomography and Correlative Microscopy. J. Neurosci..

[85] Zhang P. (2013). Correlative cryo-electron tomography and optical microscopy of cells. Curr. Opin. Struct. Biol..

[86] Sutton G., Sun D., Fu X., Kotecha A., Hecksel C.W., Clare D.K., Zhang P., Stuart D.I., Boyce M. (2020). Assembly intermediates of orthoreovirus captured in the cell. Nat. Commun..

[87] Wang K., Strunk K., Zhao G., Gray J.L., Zhang P. (2012). 3D structure determination of native mammalian cells using cryo-FIB and cryo-electron tomography. J. Struct. Biol..

[88] Watanabe Y., Mendonça L., Allen E.R., Howe A., Lee M., Allen J.D., Chawla H., Pulido D., Donnellan F., Davies H. (2021). Native-like SARS-CoV-2 Spike Glycoprotein Expressed by ChAdOx1 nCoV-19/AZD1222 Vaccine. ACS Cent. Sci..

[89] Zhu Y., Sun D., Schertel A., Ning J., Fu X., Gwo P.P., Watson A.M., Zanetti-Domingues L.C., Martin-Fernandez M.L., Freyberg Z. (2021). Serial cryoFIB/SEM Reveals Cytoarchitectural Disruptions in Leigh Syndrome Patient Cells. Structure.

[90] Zhang P. (2019). Advances in cryo-electron tomography and subtomogram averaging and classification. Curr. Opin. Struct. Biol..

